# Governance of health research funding institutions: an integrated conceptual framework and actionable functions of governance

**DOI:** 10.1186/s12961-020-0525-z

**Published:** 2020-02-18

**Authors:** Pernelle Smits, François Champagne

**Affiliations:** 1 Université Laval, Pavillon Palasis Prince, 2325 Rue de la Terrasse, Québec, QC G1V 0A6 Canada; 2 Département d’Administration de la santé, University of Montréal, 7101 Avenue Parc, Montréal, Québec H3N 1X7 Canada

## Abstract

**Background:**

Health research has scientific, social and political impacts. To achieve such impacts, several institutions need to participate; however, health research funding institutions are seldom nominated in the literature as essential players. The attention they have received has so far focused mainly on their role in knowledge translation, informing policy-making and the need to organise health research systems. In this article, we will focus solely on the governance of national health research funding institutions. Our objectives are to identify the main functions of governance for such institutions and actionable governance functions. This research should be useful in several ways, including in highlighting, tracking and measuring the governance trends in a given funding institution, and to forestall low-level governance.

**Methods:**

First, we reviewed existing frameworks in the grey literature, selecting seven relevant documents. Second, we developed an integrated framework for health research funding institution governance and management.

Third, we extracted actionable information for governance by selecting a mix of North American, European and Asian institutions that had documentation available in English (e.g. annual report, legal status, strategy).

**Results:**

The framework contains 13 functions – 5 dedicated to governance (intelligence acquisition, resourcing and instrumentation, relationships management, accountability and performance, and strategy formulation), 3 dedicated to management (priority-setting, financing and knowledge transfer), and 5 dedicated to transversal logics that apply to both governance and management (ethics, transparency, capacity reinforcement, monitoring and evaluation, and public engagement).

**Conclusions:**

Herein, we provide a conceptual contribution for scholars in the field of governance and health research as well as a practical contribution, with actionable functions for high-level managers in charge of the governance of health research funding institutions.

## Background

Research governance needs careful consideration, not only for the sake of good governance but also for the added benefits gained from an efficient health research sector in terms of the health of the population. To reinforce research governance, some actors advocate and push for the strong and explicit handling of fragmented science policy – policy-makers push for a pragmatic research agenda where there are benefits to the economy or to population groups, researchers advocate for the steering of research on health systems governance, and research organisations, such as universities and research funding institutions, decide on topics of focus and ways to attribute funds.

Health research, and research in general [1], has scientific, social and political impacts [2]. Health research performance can be measured in terms of productivity (i.e. number of papers per researcher), quality (i.e. number of highly cited papers), impact on healthcare quality, health status or the economic value of patented products (i.e. new devices) [3], and public engagement [4–6]. While there is no international consensus on the best indicators for health research [7], and there are limitations inherent to its metrics (time, attribution, etc. [8]), there is now consensus that the benefits of health research require counting, and that “*how health research systems should best be organized is moving up the agenda of bodies such as the World Health Organization*” [9].

Health research systems vary noticeably across countries, for instance, within the Western Pacific region [10], eastern Mediterranean countries [11], Latin American countries [12] or African countries [13, 14]. A comprehensive framework would provide tools to compare systems, facilitate the identification of the range of options and guide the measurement of their characteristics in order to point out ideas for complementary arrangements.

## About governance of health research by funding institutions

Health research funding institutions with a national scope encompass politics and government, advisory bodies, organisations funding research, intermediary organisations and institutions performing research, either agencies, ministries or institutes (henceforth named institutions); we refer to funding institutions of science or of health science systems that are publicly run and that cover basic and applied health research. Tetroe refers to major public research funders responsible for funding health research at the national level [15].

Few frameworks on health research systems are available. Two characteristics can be distinguished, namely governance and/or management functions. Though the ‘governance’ and ‘management’ of research might be understood and used as synonyms [16], we distinguish governance functions from those of management based on notions of internal and external environments. Following Mitchell and Shortell’s [17] typology of governance and management functions, we consider governance as being primarily concerned with positioning health research relative to the external environment in which it operates, while management is primarily concerned with daily tasks and implementation.

Broadly speaking, governance of health research “*is a framework through which institutions are accountable for the scientific quality, ethical acceptability and safety of the research they sponsor or permit*” [16].

Some frameworks might consider the health research delivery level or they may be more generic. In general, they mainly emphasise what governance or management features need to be enacted inside the organisations that deliver research, such as universities and research centres, highlighting the roles of researchers and public administrators [16, 18] or even the potential role of policy-makers [15, 19, 20]. Research funding institutions are seldom nominated in the literature as essential players in health research governance (HRG). Indeed, research on funding institutions has not received broad attention [21–24] but is slowly growing with WHO’s Health Research Policy and Systems initiative [9] and reflections on knowledge translation [25]. We will focus solely on HRG and the management of national funding institutions.

The intent of this paper is to provide an overall framework of HRG and management for funding institutions. The content is designed to support health research reformers, funding institution managers and government officials in charge of health research development. It applies to all research under the responsibilities of funding institutions, be it health services, public health, biomedical or clinical research.

We will first provide a framework of research governance and management applied to the health domain for funding institutions. We will then present international cases of funding institutions and how they enact functions and build upon case descriptions to draw some practical applications of the HRG functions for funding institutions. We finally discuss the applications for funding institutions.

## Methods

### Review of existing frameworks

Existing frameworks (Table 1) were identified via a grey literature search for all hits on Google using the following keywords: frame* OR model, combined with “Health research governance” OR “governance of health research” OR (“research for health” + “governance”) anywhere in the page. We also ran Google scholar [26, 27], searching anywhere in the article, for the first 600 hits using the following keywords in the title: “Health research governance” OR “governance of health research” OR (“research for health” + “governance”).

We excluded references that were specific to one theme, for example, genomic or epidemic, as well as those dedicated to one institutional level (e.g. university), private institutions, advocacy-oriented institutions (e.g. think tanks), or a single aspect of governance (e.g. law, ethics) or a population (e.g. librarians). We included references that were specific to public organisations (e.g. agency, ministry, institute) and the national level.

### Theoretical development of conceptual integrated framework

The methodology to develop the framework of HRG is based on the integration of the existing frameworks related to (health) research governance and to the governance of health [28].

One author of the present paper read the identified frameworks and classified the dimensions lists as per their governance, management or principles content. Whenever the authors provided a classification, we copied and pasted what they considered governance, management or principles into our documentation. When authors did not provide any specific classification, we used the definitions of governance and management used to develop the integrated framework. Governance refers to broad functions or ‘know-why’, the vision and relationships to the external environment, management refers to ‘know-what’ and operational daily tasks carried out within the environment of the institution, and transversal functions refer to ‘know-how’. Those transversal functions are, in essence, the principles that apply to governance and management functions.

### Practical application of newly developed framework to a sample of institutions

The methodology used to analyse cases was a two-step process involving the selection of countries (Table 2) and institutions (Table 3). We sought research funding institutions from a diversity of countries. The selection of countries rests upon the acknowledged leadership in English-speaking health research production and a mix of North American, European and Asian countries.

**Table 1:** List of selected frameworks

Name of the institution	Name of the framework
▪ Canadian Institutes of Health Research (CIHR)	Health research and health-related data – Framework and action plan
▪ Council on Health Research for Development (COHRED)	COHRED research and innovation for health system development framework
▪ European Observatory on Health Systems and Policies	TAPIC framework (transparency, accountability, participation, integrity, capacity)
▪ National Institute for Health and Care Excellence (NICE)	NICE research governance framework
▪ Health Research Authority & Departments of Health United Kingdom	United Kingdom policy framework for health and social care research
▪ National Health and Medical Research Agency (NHMRC) Australia	Research governance framework
Name of authors	Name of the framework
▪ Pang et al. [33]	Conceptual framework and foundation for health research systems
▪ Rani et al. [10]	Health research: essential governance and management functions

**Table 2:** List of selected countries

Canada	Sweden
United Kingdom	The Netherlands
Australia	Singapore
United States of America

**Table 3:** List of selected institutions

Canada	Sweden
Canadian Institutes of Health Research (CIHR)	Swedish Research Agency/Vetenskapsrådet – Scientific Council on Medicine (SRC)
United Kingdom	The Netherlands
National Institute for Health Research (NIHR)	The Netherlands Organisation for Health Research and Development (ZonMW)
Australia	Singapore
National Health and Medical Research Agency (NHMRC)	National Medical Research Agency (NMRC)
United States of America	
National Institutes of Health (NIH)

**Table 4:** Matching dimensions of health research governance

C1	C2	C3	C4	C5	C6	C7	C8	C9
Canadian Institutes of Health Research (CIHR)	Council on Health Research for Development (COHRED)	European Observatory on Health Systems and Policies	United Kingdom	National Institute for Health and Care Excellence (NICE)	National Health and Medical Research Agency (NMHRC)	Pang et al. [33]	Rani et al. [10]	Our integrated framework
Related to governance								
• Open access • Proactivity of needs and opportunities	• Ensure political and socioeconomic climate • Financing • Priorities • Information systems • Collaboration and partnerships	• Accountability		• National legislations and policies (roles and responsibilities, research malpractice)		• Define and articulate vision • Identify priorities • Coordinate adherence to priorities	• Identifying and pursuing national priorities • Oversight of health research • Developing and pursuing national research strategies	• Intelligence acquisition, • Resourcing and instrumentation • Relationship management • Accountability and performance • Strategy formulation
Related to management								
• Resource, skills, access • Sustainability of data • Discoverability (visibility and use of data)	• Monitoring and evaluation • Communication • Contracting • Coordination	• Policy capacity to align with resources in pursuit of goals	• Responsibilities of individuals • Responsibilities of organisations	• Notification of high-risk projects • Agreement to participate • Methodological review (peer review, review of existing research) • Expertise and good clinical practise • Information confidentiality and good practice	• About a proposed research project • Initial assessment of research • Final assessment • Risk management • Assessment of legal and administrative requirements • Credentialing and supervision • Authorisation • Monitoring complaints management • Reporting completion	• Monitor and evaluate health research systems • Financing: secure funds and allocate • Create and sustain resources and capacities • Produce, translate, promote research	• Monitoring overall research activities • Monitoring, building, strengthening and sustaining national health research capacity • Creating systems to facilitate wider access and dissemination	• Priority-setting • Knowledge transfer • Financing
Principles								
• Integrity of data • Respect right, ethics, culture • Value (intellectual property, etc.)	• Ethics and review • Community and civil engagement	• Transparency about decision, process and grounds • Participation of affected parties • Integrity of process clearly specified	• Safety, competence, scientific and ethical conduct • Patient, service users and public involvement, choice, insurance and indemnity, respect for privacy • Integrity, quality, transparency, compliance • Protocol • Legality • Benefits and risks • Approval • Information about the research • Accessible findings • Justified interventions, ongoing provision of treatment, integrity of the care record, duty of care	• Service users, public participations • Protecting research participants • transparency	• Intellectual property arrangements • Ethical review	• Set and monitor ethical standards	• Ethical conduct	• Transparency • Capacity reinforcement • Monitoring and evaluation • Ethics • Public engagement

**Table 5:** Brief description of features of funding institutions

Countries	Canada	Australia	United States of America	Singapore	Sweden	United Kingdom	The Netherlands
Name of institution	Canadian Institutes of Health research (CIHR)	National Health and Medical Research Agency (NHMRC)	National Institutes of Health (NIH)	National Medical Research Agency (NMRC)	Swedish Research Agency (SRC) –Scientific Council for Medicine^a^	National Institute of Health Research (NIHR)	The Netherlands Organisation for Health Research and Development (ZonMw)
Date of constitution act	2000	1992	1930	1994	NA	2006	2001
Budget, yearly (equivalent in euros^b^)	CAD$1102.9 million CAD $ budget in 2017–2018 (€ 760 million)	AU$800 million 2016–2017 (€ 500 million)	NA	US$492.7 million budget in 2016 (€ 450 million)	570 million SEK budget in 2006 on health and medical research (€ 53 million)	£207 million pounds budget in 2016–17 (€ 240 million)	NA
Volume of projects/or researchers and trainees supported	13,700 researchers and trainees in 2015	1035 grants (not grantees)	10,000 research project grants	1100 individual research projects	420 projects approved in 2006	263 projects	NA
						5500 researchers annually	
			In 2014, 35,000 principal investigators				
Investigator-initiated grants/targeted funding	70–30%	Management of some priority-driven funding schemes of the Medical Research Future Fund perpetual fund	NA	1/3–2/3	Mostly investigator-initiated grants	Commissioned and research led	Targeted calls for Health Care Efficiency Research programmes
Thematic institutes/centres	13	none	27	0	In the form of council	0	0

^a^Sweden has no institute dedicated solely to the health and medical sector. The SRC is the main contributor to R&D, including for health research. Other national sources also contribute to health research. For example, the Swedish Research Council for Health, Working Life and Welfare funds research on public health and the public health system [39]. The Swedish Research Council for Health, Working Life and Welfare (Forte) distributed around US $55 million in 2014 [40]

^b^In current dollars, 2019

*NA* not available

**Table 6:** About intelligence acquisition across funding institutions by case

Country, institution	Actions related to intelligence acquisition
Canada, Canadian Institutes of Health Research (CIHR)	• CIHR shall answer to the minister’s request for advice (*CIHR Act*)
	• Achievements of CIHR available in implementation reports, audit reports, international reviews
	• Input from scientific directors of institutes for the strategy of CIHR
	• Archives on reports of CIHR, minutes of board
	• Published management response to reviews of programmes
	• Initiatives and tri-council fund across institutes
	• Seek inputs from research community on mission achievements
Australia, National Health and Medical Research Agency (NHMRC)	• Pre-established profile of expertise for institution members depending on health domains
	• Targeted call for research drafted by NHMRC alone or on demand from Australian health authorities
	• Participation in developing national strategies for research infrastructure investment
United States, National Institutes of Health (NIH)	• Office of Legislative Policy and Analysis (OLPA) to track bills, laws, hearings, legislative updates, congress committees of interest to NIH
	• Upon director’s request, input on strategy development come from the Council of Public Representatives (COPR)
	• For emerging opportunities and rising health challenges, the division of programme coordination, planning and strategic initiatives accelerates investments
	• Excel by managing for results (bibliometrics, dynamic model of workforce, etc.)
Singapore, National Medical Research Agency (NMRC)	• Revisiting policies with the inputs from researchers, research offices of institutions, finance and human resource personnel
	• Engagement to give feedback on suggestions that are rejected in development and revision of policies
	• Research around top-down direction from Ministry of Health
Sweden, Swedish Research Agency (SRC) and Scientific Council for Medicine (SCM)	• Majority of board members of SRC and SCM elected by the research community
	• Assessment of Swedish participation in EU framework programme
	• Evaluation of 20 research environment funded by Linnaeus grant
	• Monitoring of research policy development (bibliometric studies)
United Kingdom, National Institute of Health Research (NIHR)	• Multiple partnerships with private and charity organisations
	• Industry liaison team at NIHR
	• Support for evidence-based medicine and evidence-based policy-making (health technology assessment, Cochrane Centres, etc.)
The Netherlands, The Netherlands Organisation for Health Research and Development (ZonMw)	• Evaluations of programmes and of ZonMW
	• Programme committee investigates the urgency and relevance of researches proposed by the ministry
	• Two institutions head ZonMW
	• In 2015, undertook wide consultation of civilians, companies, researchers and social organisations to provide questions to the scientific community, with attached roadmaps

**Table 7:** About strategy formulation across funding institutions by case

Country, institution	Actions related to strategy formulation
Canada, Canadian Institutes of Health Research (CIHR)	• Strategic plan of CIHR available
	• Mission and values of CIHR and institutes available
	• Representation of citizens on board of CIHR and some institutes
	• Involvement of institutional partners in health (health ministry, etc.) to set up priority themes of institutes
Australia, National Health and Medical Research Agency (NHMRC)	• Strategic direction available online
	• Open invitation online to comment a proposal for a specific strategy
	• Chief medical officer/chief health officer for each state and territory sit on the institution CEO identifies major national health issues
United States, National Institutes of Health (NIH)	• Each institute and centre has a strategic plan available
	• Some interagency initiatives with strategic plans
	• NIH with roadmap on 21st century vision
	• NIH priority set by scientific community, institutes and centre advisory agency, public, congress and administration, others
Singapore, National Medical Research Agency (NMRC)	• Mission statement available
	• 1 multidisciplinary local review panel
Sweden, Swedish Research Agency (SRC) and Scientific Council for Medicine (SCM)	• Online strategy
	• Involvement of the research community in strategy formulation through elected university members of institutions and SRC board
United Kingdom, National Institute of Health Research (NIHR)	• Online statement
	• NIHR advisory board with leaders from academics, patient and public representatives to give advice on strategic development
	• Translation research under a cooperation with Medical Research Council, coordinated by a new Office for Strategic Coordination of Health Research
The Netherlands, The Netherlands Organisation for Health Research and Development (ZonMw)	• Government and policy-makers contribute to the discussions and decisions of the advisory committee for eligible programmes to fund

**Table 8:** About resourcing and instrumentation across funding institutions by case

Country, institution	Actions related to resourcing and instrumentation
Canada, Canadian Institutes of Health Research (CIHR)	• Internal policies and documents on insurance coverage for CIHR volunteers, Personnel Security Screening Policy, CIHR Travel Policy, CIHR Travel Expenses Reimbursement Guidelines, CIHR Code of Conduct
	• Collaborations with partners (e.g. the SPOR strategy with pharmaceutical companies)
	• Website for partnership solicitation
	• Financial support of CIHR Board to support institutes’ roles
Australia, National Health and Medical Research Agency (NHMRC)	• Assigns academy member group to identify external reviewers and to monitor peer review
	• Provides acceptable standards to research institutions on intellectual property, ethics and responsible conduct of research
	• Added some specific committees upon the request of the Minister of Health and Ageing
United States, National Institutes of Health (NIH)	• Open application process for members of the Council of Public Representatives (COPR)
	• Allocation of institute funds for activities under the institute’s control
Singapore, National Medical Research Agency (NMRC)	• Revised financial and administration guidelines for researchers
	• Internal policies not online in English
	• Guidelines on data sharing, guidelines for media
Sweden, Swedish Research Agency (SRC) and Scientific Council for Medicine (SCM)	• Two basic research-targeted programmes on Swedish priority research topics
	• Organised a series of seminars and a 1-day conference on mobility of researchers in the EU
	• Digital service to communicate with journalists
	• Measurement of citation impact of Swedish research
	• Open access to research data
	• Research data registry
United Kingdom, National Institute of Health Research (NIHR)	• Research capability funding to support the management of research funds
	• Research support services as NIHR service to encourage, frame and harmonise local research processes for NHS-funded research
	• Development of unified approval process, standards for compliance
	• National advice service for complex regulatory issues of local R&D, with partners
	• Partner with industry to support private economic growth
	• Development of core standards for public involvement
	• Plain English award
	• Consensus building on the interpretation of central policies and rules by local R&D offices of NHS trusts
The Netherlands, The Netherlands Organisation for Health Research and Development (ZonMw)	• Minutes of committee meetings – details not available
	• Responsibilities of committee members – details not available
	• Policies of committee – details not available

**Table 9:** About management of relationships across funding institutions by case

Country, institution	Actions related to management of relationships
Canada, Canadian Institutes of Health Research (CIHR)	• Internal relationships with the scientific directors of institutes that have a seat at the CIHR scientific institution
	• University delegates to represent CIHR on campus
	• Interaction with Parliament in 2005, with Day on the Hill
	• Speak with one centralised voice for CIHR and its institutes
	• New initiatives to connect with other institutions and institutions (Natural Sciences and Engineering Research Council of Canada and Social Sciences and Humanities Research Council of Canada)
	• Declaration of conflict of interest on governing institution board meetings
Australia, National Health and Medical Research Agency (NHMRC)	• Involvement of funded researchers in research translation faculty to advise NHMRC on knowledge translation
United States, National Institutes of Health (NIH)	• NIH and each institute with a public liaison office
	• Council of Public Representatives to advise NIH director on public participation, bringing public voice and sending messages to public
	• Seeking out partnerships across institutions and with private actors to enhance the impact and translation of findings
	• Pioneering a proactive participation model with the inclusion of volunteers at all stages of the research process
Singapore, National Medical Research Agency (NMRC)	• Public engagement programme, website, education material, public forum to inform on clinical research activities and present the need for active participation on clinical research and trials
Sweden, Swedish Research Agency (SRC) and Scientific Council for Medicine (SCM)	• Advise government on research policy
	• Collaboration with a book fair where universities and higher education institutions interact with a broad audience
	• Strengthening community (local teachers/schools); support of the EU initiative Researchers’ Night
	• Broad information programme for public and decision-makers
	• Influence on research policy agenda by publishing on research policy in Sweden
	• Responsibilities for EU programme Science in Society
	• Mission to advise the government
	• Web service to connect journalists and experts
	• Implication in EU framework (viewpoint on the design of the call for application, ERA-NET programme, promotion of pan-European calls for proposals)
	• Development of collaboration with Nordic countries on research infrastructure
	• Hosting of an international conference on research communication in 2008
	• Annual meeting for researchers and politicians
	• Breakfast seminars at the parliament with researchers
United Kingdom, National Institute of Health Research (NIHR)	• NIHR network to connect researchers and experts in specific domains (share good practice, study feasibility, etc.)
	• Public involvement in commissioning and reviewing of proposals by coordinating centres
	• Facilitation of industry engagement with office for clinical research infrastructure
	• Connecting with a newspaper to publish clinical trials and number of patients recruited
	• Secretary of State for Health’s visit to a research centre
	• Biannual conference on public involvement with public/researcher/organisation
	• Showcase of R&D work opened to the public
	• Research output assessment tools to capture progress of commissioned research
	• Launch of a press office in2017
	• Initiative INVOLVE to promote and advance public engagement in research
The Netherlands, The Netherlands Organisation for Health Research and Development (ZonMw)	• Collaboration with the EU on different projects
	• Collaboration at international level on specific programmes (i.e. Heads of International Research Organisations)
	• Practice-oriented design and assessment of research proposals with patients, city councils, etc.

**Table 10:** About accountability and performance across funding institutions by case

Country, institution	Actions related to accountability and performance
Canada, Canadian Institutes of Health Research (CIHR)	• Criteria for the selection process of board members online
	• Accountable to parliament through the Ministry of Health
	• Resignation procedures in by-law
	• Regular public reports: annual audit on finance, activities, quintennial review on management and governance
	• Stakeholders involvement in review
	• Review process of CIHR or its components with an independent international panel
	• Online disclosure of reports, expenses, law and by-laws
	• Online composition and terms of reference for committees
	• CIHR code of ethics
	• Limitation of consecutive mandates
	• Regular reports on performance (CIHR audits, CIHR reviews, programme evaluations)
	• Online decisions about the attribution of grants and committee members
	• Planned performance indicators mentioned in roadmap
Australia, National Health and Medical Research Agency (NHMRC)	• Report on annual performance reached with a set of targets
	• Proceedings of agency meetings online
	• Commissioner of complaints
	• Online list of names of members of committees
	• Internal audit committee
	• Emphasis on integrity as an objective of strategic direction 2015–2019, and as a central activity of NHMRC in providing standards and guidelines
United States, National Institutes of Health (NIH)	• Online diffusion to the public (news radio, public lecture, free film festival)
	• Develop methodology to evaluate scientific investment
	• Plans for review of peer-review process, administrative burdens
	• Series of initiatives to enhance conduct of science
Singapore, National Medical Research Agency (NMRC)	• Annual report with financial data, projects and individuals funded
	• Internal procedures and activities not online
Sweden, Swedish Research Agency (SRC) and Scientific Council for Medicine (SCM)	• SRC investigates fraud in attribution of funding and manipulation of science results
	• Sweden and international reviewers on peer-review grant applications
	• Online policies and SRC viewpoints
	• Online decisions about the attribution of grants
United Kingdom, National Institute of Health Research (NIHR)	• Duty of Secretary of State for Health, NHS commissioning board, clinical commissioning groups in respect of research are written in Health and Social Care Act 2012
	• Terms of reference and memberships of advisory board are online
	• Terms of reference and membership of strategy board available online
	• Relationships between research and improved quality of care to be reported based on an illustrative model statement
	• Results of training competition are online
	• Funding to NHS service providers have been conditional to professional management of health research since 2012
	• Agenda and minutes of advisory board are online
	• Publication of NIHR-funded recipients online
	• NIHR benchmarking of recipients of clinical studies
	• Plan to publish NIHR trusts-level performance comparisons
The Netherlands, The Netherlands Organisation for Health Research and Development (ZonMw)	• A retiree leads each programme committee
	• List of committee members – not available
	• List of grantees – not available
	• Evaluation reports online

**Table 11:** Summary of operational dimension of governance of health research for funding institutions

Functions of governance	Salient dimension
Intelligence acquisition	• Top versus bottom influence of outsiders
	• Proactive versus reactive knowledge hunting
	• Presence versus scarcity of organisational learning procedures
Strategy formulation	• Research insider versus research outsider
	• Single health sector versus multiple sector inputs
Resourcing and instrumentation	• Providing support material for the entire research community versus restricting it to grantees
	• Providing open grant versus targeted grant
	• Pushing or not for linkages to healthcare benefits
	• Questioning or not competitiveness in a globalised research environment
Management of relationships	• Internal versus external partners
	• Customary versus recurrent partnerships
Accountability and performance	• Disclosure of process or results
	• Follow-up on diverging behaviour or not
	• Committee related information online or not

The criteria for being a major provider of research funds were being funding institutions from the public sector, national in scope, funding health-related research and being a major provider of research funds. A team composed of professors, researchers, consultants and managers from funding institutions and research centres (total of 6 individuals; 2 from the field of governance, 1 finance, 1 academic training, 2 international management, equally coming from academic and practical background; 4 of these directly worked with funding institutions) selected the cases.

The information included in this study was extracted from documentary sources, including reports of the selected funding institutions available as of November 2018 (annual report, strategic plan), related strategic information whenever available from the website of the selected funding institutions as consulted in November 2018 (e.g. organisational chart, procedures, mission), and the legal status of the selected funding institutions (i.e. the constitutive act in force) (See Appendix – data sources for further details). Some institutions documented their strategy and actions at much more detailed levels than others; we considered what was mentioned independently of the level of detail.

One member of the study team read through all documentation, and then extracted and classified information relevant to the stated dimensions of the framework (Tables 6, 7, 8, 9 and 10). A round of verification and collection of complementary data took place by sending a request to each institution for comments from the direction of communication, cc’d to the contact of the head manager of each funding institution. Out of seven institutions contacted, we received three answers. The institutions were asked for the following information: (1) to complete information about their institution, and (2) to comment on the validity of the five governance-related dimensions (e.g. Do they make sense to you? Are they clear? Anything missing?).

## Results

### Brief review of the existing frameworks

A national framework on HRG outlines the understanding of a government about its vision of health research, internal and external roles, and the philosophy behind running high-standard health research. It is a formal statement on how to improve research and safeguard the public [29]. It gives clear directions on what to work on and how to practice efficiently in order for the population to benefit from health research results and new knowledge. Such frameworks eventually include people, institutions and activities, and enable the health research system to generate and use knowledge for the benefits of health. A framework provides a systematic tool to portray the health research system in a systematic manner [30].

At least eight recent frameworks on health research are available – the Department of Health in the United Kingdom published a framework that gives details on standards and responsibilities for health research [31]; the Council on Health Research for Development (COHRED) developed a framework with technical components of particular aspects of health research systems [32]; Pang et al. synthesised a consultation on the foundation for health research systems [33]; Rani et al. presented the governance and management functions extracted from a consultation in low- and middle-income countries [10]; the Canadian Institutes of Health Research (CIHR) offers principles and stewardship details for the collection and use of data overall [34]; the European Observatory mainly provides a set of principles that can be divided into managerial and governing mechanisms [35]; and, finally, the Australian Research Council sets a step-wise framework to manage research projects [36].

Some frameworks focus more on research governance for research institutions (universities, etc.), others encompass research governance for funding institutions. Indeed, the National Institute for Health and Care Excellence (NICE) and the National Health and Medical Research Agency (NHMRC) frameworks focus extensively on the aspects that need to be considered by an institution receiving NICE or NHMRC funds. In these frameworks, dimensions are closer to a set of steps to be filled from the inception to the closing of a research project. All other frameworks refer to governance (sometimes called “*stewardship*” by Pang et al. [33]), management and a set of more or less detailed principles. Explicit concerns for ethics and public participation are prevalent among these principles (see Table 4, columns C1 to C8).

Some frameworks provide an overarching set of dimensions, whereas others delve into the specifics of either management or governance. Indeed, the COHRED and European Observatory frameworks are both designed as overarching frames, covering multiple dimensions. In the case of COHRED, 15 dimensions provide many details on principles for managerial- or governance-related aspects. The European Observatory framework similarly gives a broad view of what dimensions to consider, although it condenses the number of dimensions down to five.

The European Observatory framework appears as the most overarching framework. Each of the principles proposed is accompanied by a set of specific mechanisms that help those in charge of governance or managerial functions to act accordingly. For instance, the principle ‘accountability’ includes mechanisms for managerial functions, such as competitive bidding, and some mechanisms for governance purposes such as conflict of interest policies and codes of conduct.

The COHRED framework is based on ‘key aspects’ of health research and has ‘action guidelines’ attached to each of them, covering governance and management functions. Key aspects include a conducive environment for ethics and leadership, a solid base of policies, priorities and management, and the ability to perform and produce in the areas of resources, optimisation and international integration. It is formatted in the spirit of a step-by-step guide to improving research governance at the national and institutional levels. It lists good practices and advice such as formalising partnership arrangements and ensuring transparency through the ranking process.

Pang et al.’s [33] framework builds four functions. One essential pillar is ‘stewardship’, whereby vision, priorities and monitoring provide direction for health research. ‘Financing’ makes it possible to get funds in and to allocate funds with accountability; the ‘creation and sustainability of human and physical resources’ and ‘the production and use of research’ complete the framework. Note that production and use of research belongs to both the governance functions and the management functions categories if organisations are performing research and knowledge transfer. Accountability is related to financing.

Rani et al. [10] propose essential governance and management functions based on consultation with low- and middle-income countries, advocating for the improvement of ethics committees and of registries to record funding and research data.

The CIHR framework is organised into five main functions of governance. As the framework relates both to health research and health-related data, the dimensions reported have a digital flavour, focusing on data quality, open access, data visibility and so forth. The transfer of these five broad guiding principles and five components (vision, culture, resources, skills, access) can easily apply to organisations and systems running research projects, right up to health research governing bodies such as funding institutions. This framework is particularly concerned with reaching out to all involved stakeholders and with compulsory actions, specifying who is responsible and what activities have to be checked and approved.

The NICE framework is particularly concerned with each and every person working at or for NICE itself, clarifying the roles, responsibilities and institutions to contact in different scenarios.

NHMRC’s Australian framework provides a roadmap for those organisations and systems running research projects who need to comply with high-standard research governance.

All the above frameworks seem relevant for a funding institution. In the following section, we propose an integrated framework. Dimensions that were cited by others are integrated into the encompassing governance and management HRG framework that we propose below. We distinguish which functions are more closely related to management functions and which are more closely related to principles or governance.

### Conceptual integrated framework on governance and management of health research by funding institutions

**Fig. 1:**
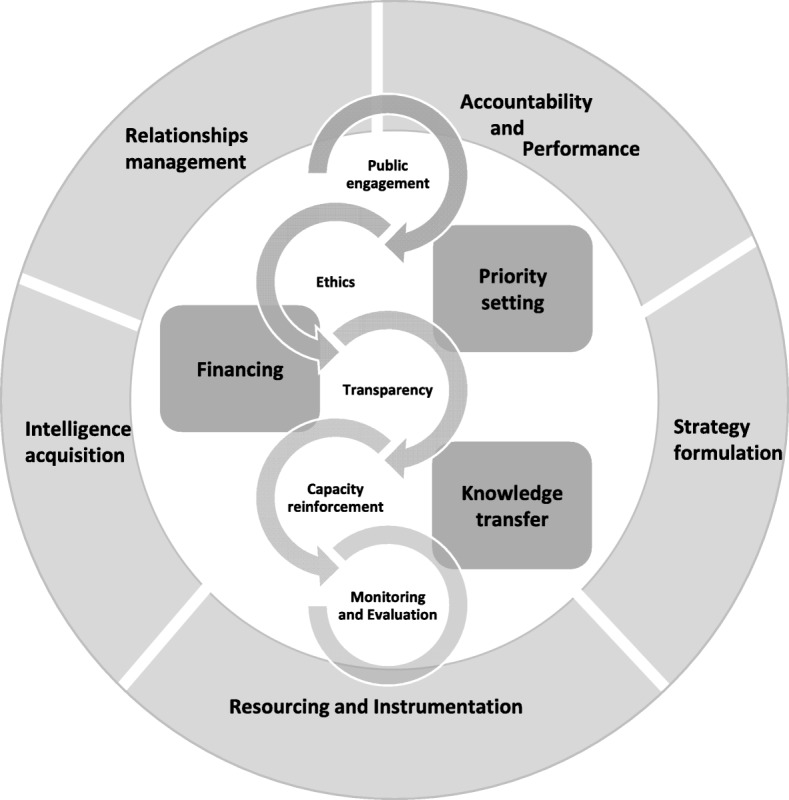
Framework on governance of health research

We propose to build the Framework on Governance of Health Research (FGHR) upon these existing frameworks (Table 4, column C9). FGHR also grows out of our understanding of governance in health research and health systems, our observation of governance practices in health research and health systems, and the inputs from the above frameworks. We acknowledge that, at times, delimitations might be blurry between governance and management functions. Therefore, we decided to organise the FGHR around three groups of functions (governance, management, transversal functions), as presented in Fig. 1. Here, governance is shown on the outside of the figure, representing broad functions (or know-why), management functions (or know-what) are shown inside the circle and are run within the standards set by governance and some transversal encompassing functions are present in both governance and management levels (or know-how).

The composition of FGHR reflects governance functions, management functions and transversal functions. Governance functions reveal the steering activities that actors and institutions must undertake to ensure a fit between the health research system and the external environment. Management functions correspond to activities to be carried out internally on a daily basis to ensure the pursuit of health research for funding institutions, universities, research centres and principal investigators. Transversal functions qualify management functions and the effects required from the actualisation of governance functions. The term refers to good practices and excellence in the exercise of management and governance in health research, namely transparency, capacity-building, monitoring and evaluation, and ethics.

FGHR is composed of five governance functions, three management functions, and four essential types of know-how. The framework’s five governance functions are ‘intelligence acquisition’, ‘resourcing and instrumentation’, ‘relationship management’, ‘accountability and performance’, and ‘strategy formulation’. Intelligence acquisition is the production and acquisition of the knowledge necessary for providing a vision for the health research that the organisation supports and for the consultation and recruitment of adequate expertise. Resourcing and instrumentation refers to the acquisition and generation of the means to achieve strategic goals through board meetings, reports and reviews, inward flow of monetary resources, and the means to support the development of governance structures and activities such as explicit responsibility and task descriptions. Relationship management is concerned with ensuring good and efficient connections, both with the external environment and internally with insiders such as the direction committee. Accountability and performance relate to the ability of the organisation to exercise good governance through instituting the means to track its own development and activities as a governance structure. This function relates to a reflexive capacity of governance. Formulating mission and vision is the process of setting up the strategic content, mission, vision and priorities with adequate policies and ethical codes to exercise governance functions.

The framework’s three management functions are ‘priority-setting’, ‘financing’ and ‘knowledge transfer’. Priority-setting refers to the process of setting up midterm actions that match the vision of the organisation. Financing refers to the outward flow of monetary resources as funds are allocated. Knowledge transfer covers the organisation’s support for knowledge-transfer activities. It can be organisation-led, such as the funding institution facilitating meetings between the scientific community and politicians, or related to research funding, whereby researchers can apply for specific knowledge-transfer grants.

The five transversal functions of the framework are based on essential types of know-how underlying HRG and management; they are ‘ethics’, ‘transparency’, ‘capacity reinforcement’, ‘monitoring and evaluation’, and ‘public engagement’. Ethics refers to the quality of a process, either governance functions or management functions in the selection of board members or in the attribution of grants through peer-review processes. Transparency refers to the disclosure of procedure and results, for example, having clear and publicly available criteria for election to boards and committees, posting the names of successful research grant applicants online, or providing free access to publications. Capacity reinforcement relates to a continuous organisational effort to support the development of human resources, in terms of either board members or staff employed by the organisation in a management function as well as the support for capacity development when funding students. Monitoring and evaluation cover processes of data collection and analysis to follow-up on, estimate the performance of, and benchmark organisational processes and results. Public engagement refers to efforts to reach out and/or integrate the population or groups of the population in an authentic and continuous decision-making process.

This FGHR intends to establish principles for carrying out health research at the national level. The scope of the FGHR covers the responsibility of the public system for the governance of health research – from the top-level decision-making organisations that fund research to the recipient organisations that implement research projects in health domains. The framework is directly relevant to those who target, fund, manage, host, conduct, participate and accredit health research. It can theoretically apply to all health research related to studies sponsored by the ministry level, to research carried out within a geographical area, and to research funded totally or partially with national-level public funds.

The framework seeks to establish the essential functions and values of health research conduct. Existing requirements binding research communities or existing laws and requirements designed to protect research participants, to ensure confidentiality, and so forth, are not integrated at this point. The responsibilities of institutions and actors can be defined in future steps.

These governance functions do corroborate some of the governance tasks for research policy and practice in health mentioned by Mitchell and Shortell’s [17], namely obtaining financial resources and providing measures for accountability.

### Practical application of the newly developed framework in terms of governance

We further refer to actionable functions as useful actions [37] that bring clear directions [38] to enact governance. We decided to focus solely on governance functions because much is already written on management and ethics in research.

#### Description of the research environment by case

Each year, funding institutions individually invest between US $90 million and US $31 billion in health research to fund researchers, trainees and projects. Some countries, such as Canada and the United States, organise their budget around thematic research organisations and some countries flag available funding on thematic studies rather than organisations (Table 5).

A direct comparison between funding institutions is difficult to establish, with some reporting the prevalence of researchers and trainees currently supported on a yearly basis, others the incidence of researchers and trainees newly funded during the year. A wide diversity prevails in terms of funding models. CIHR in Canada favours investigator-initiated grants, whereby researchers nominate a topic of research in which they are proficient and for which they would like to receive funding. In Singapore, the opposite dynamic seems to prevail, with the majority of funds dedicated to targeted grants on specific topics of interest to the government. Our main intent in presenting several cases is to provide a practical look at various governance frameworks and to extract empirical applications.

#### Analysis of governance functions in health research funding institutions by case

- Intelligence acquisition

‘Intelligence acquisition’ refers to the means put in place by funding institutions to acquire their strategic knowledge and expertise. The design of funding institutions’ strategic actions might be influenced by the policy domain, for example, by government authorities or ministries. In such a situation, these inputs come from a logic of top-down representative democracy. A mixture of bottom-up inputs also seems to be widespread in funding institutions, with the participation of direct and indirect beneficiaries of funded health research; indeed, patients, the public and researchers do contribute their share of knowledge to formulate, comment on or format policies.

While funding institutions do receive some inputs, they might also look for information directly relevant to their mission as it emerges. To do so and remain open to environmental opportunities, a proactive structure might be put in place to investigate early policy developments of interest to the institution, as is done in the Netherlands (Table 6).

The mobilisation of external knowledge may be complemented by knowledge acquisition on the internal processes of a funding institution. In so doing, the institution presents a strong signal that it is a learning organisation willing to adjust as needed. Internal reviews provide evidence on which to build a continuous improvement dynamic, both within the funding institution and for its external partners. The National Institutes of Health (NIH) explicitly places a high priority on learning processes – its strategic plan proposes that it will excel as a federal institution, it reviews its peer-review processes, uses bibliometrics to indicate the value of a programme, conducts PhD workforce analysis so as to better predict the optimal number of fellows the NIH can support, and reduces the administrative burden by distinguishing between unavoidable burdens and those that are merely due to custom or habit.

As a conclusion to the dimension of intelligence acquisition, one operational and empirical application would be to consider the following aspects:

- Top-down versus bottom-up influence of outsiders
- Proactive versus reactive knowledge hunting
- Presence versus scarcity of organisational learning procedures

b) Strategy formulation

‘Strategy formulation’ refers to the exchange processes that guide the actions of founding institutions. It can take the form of developing founding documents and principles. The evidence to feed such long-term and structural decisions comes from insiders from the health research system, researchers, academics, health ministry representatives, and so forth. It might also derive from the ultimate beneficiaries of health research (citizens) and those outside the system (congress members, etc.). Another difference between the funding institutions that are developing their long-term vision, mission and policies is their openness and integration of non-health-related actors and whether it is solely focused on the health sector or not. Some institutions call for medical providers and health institutes to collaborate on the design and elaboration of a strategy. However, because the health sector opened up decades ago to the wide range of determinants of health, it is now well established that the health of the population is largely dependent on interventions made in sectors that do not fall under the jurisdictions of health ministries. Therefore, the involvement of non-health-labelled institutes and representatives is or has to be considered by funding institutions; for example, Australia’s funding institution opens its strategy to online commentary from any sector (Table 7).

As a conclusion to the dimension of strategy formulation, one operational and empirical application would be to consider the following aspects:

- Research insider versus research outsider
- Single health sector versus multiple sector inputs

c) Resourcing and instrumentation

‘Resourcing and instrumentation’ refers to the tools that are put in place to finance, fund and support the development and implementation of an institution’s strategy. Financing is the act of collecting and receiving money to run the institution; the sources of money can be public and/or private. The NIH, for example, is much closer to the private sector than other institutions portray themselves to be. Instrumentation, such as guidelines and policies, is developed for the internal functioning of an institution; for example, the description of selection criteria for committees. Online tools might also be available to support research external to the institutions, for example, the guidelines for university ethics committees as provided in Australia. Support given to researchers might be facilitated through open resources where researchers compete on broad-spectrum grants or be targeted to the needs of some government agenda or ministry priority, as happened in Australia when the then Minister of Health and Ageing requested additional committees (see Table 8). The organisational processes involved in providing money to universities, grantees, scholars and research centres – both public and private – so as to implement institution programmes through projects funded can be closely informed to reframe funding schemes. To encourage high standards of research, and highly competitive researchers, institutions look at ways to move forward in a globalised research environment and to support researchers accordingly. Sweden, for example, is reflecting upon researchers’ mobility. To align with institutions’ missions to bring value to the population and improve health, institutions such as those in the United Kingdom, propose a model of reporting in which care is explicitly taken to use plain English in order to favour clear communication of funding applications (Table 8). In this way, institutions encourage both international engagement and the translation of research results into health practices.

As a conclusion to the dimension of resourcing and instrumentation, one operational and empirical application would be to consider the following aspects:

- Providing support material for the entire research community versus restricting it to grantees
- Providing open grants versus targeted grants
- Pushing or not for linkages to healthcare benefits
- Questioning or not competitiveness in a globalised research environment

d) Management of relationships

‘Management of relationships’ refers to the preoccupation with interacting in meaningful and constructive ways with the institutions’ partners – be they insiders of the institution, such as the heads of the constituting institutes of an institution at CIHR Canada, or outsiders such as politicians or institutions unrelated to the health sector.

Some institutions run activities and set seats on boards for their internal partners (CIHR scientific agency, see Table 9). They might also connect with outside funding institutions to set up multi-institution funding for innovations or grants covering boundary work and transversal research. Such efforts to build up complementary programmes and to invite collaborators might be customary or recurrent. Over time, such recurrent relationships and exchanges with outsiders become institutionalised in the institution processes. They might also be at the pilot-testing phase or in an early development stage, when institutions establish bridges with partners on a more intermittent basis. In 2005, CIHR in Canada organised a pilot project with parliamentarians named ‘Health Researcher’s Day on the Hill’, and planned to send newsletters to members of Parliament three times a year since 2012 [41]; in Sweden, researchers and politicians are convened to a shared event on a yearly basis.

As a conclusion to the dimension of management of relationships, one operational and empirical application would be to consider the following aspects:

- Internal versus external partners
- Intermittent versus recurrent partnerships

e) Accountability and performance

‘Accountability and performance’ is the process by which a funding institution follows its own development and activities and is reflexive about its governance capacity. Because funding institutions might or do oversee the quality and integrity of the research they are funding, some have developed procedures to ensure high standards for research quality. Follow-up on research quality can take the form of inquiry into fraud in attributions of funding and the manipulation of results. Sweden, stricken by the Macchiarini case on gross scientific misconduct [42], installed a Research Misconduct Board to address such issues. Publishing information online regarding, for example, who sits on committees, who receives funds, and what type and amount of funding is received is another transparency mechanism employed by funding institutions such as CIHR in Canada and the NHS in the United Kingdom (Table 10).

Additionally, what happens behind the closed doors of granting committees might take different forms. It might address the internal processes of committees, their selection criteria or their mandates, or it might address the committee’s final decision regarding the list of grantees. An institution might therefore focus more or less on disclosing its internal procedures or on its committees’ final decisions.

As a conclusion to the dimension of accountability and performance, one operational and empirical application would be to consider the following aspects:

- Disclosure from process to results
- Follow-up on diverging behaviour or not
- Whether to put committee-related information online or not

In conclusion, we extracted a few specific operational dimensions salient to the governance of health research by funding institutions (Table 11).

## Discussion

We would like to discuss the validity of the framework for governance of health research funding institutions.

One could argue that the framework is not valid because it is based on a limited set of existing frames. Here, it is assumed that a sample is sufficient for the identification of elements of governance. A framework can be developed from a deductive approach, mobilising a catalogue of theories and knowledge from scholars. It can also be developed from an inductive approach, this time mobilising hands-on knowledge from the institutions themselves. We mainly borrowed from both approaches to develop the integrated framework, being rooted in practice, and also keeping an open door to the approaches of scholars who might have previously developed deductive frameworks. The literature refers to publications by Rani et al. [10] and Pang et al. [33], both of which use practitioners’ consultations to draw their framework.

The strength of the integrated framework will also rely upon developing it on high variability cases, including internal variability among institutions and external variability among the institutions’ national environments. The selected institutions of this study cover all health research topics rather than simply topics that fall under unique categories of medical research (e.g. stem cell), social sciences and humanities (e.g. management of primary care), or engineering (e.g. radiation therapy); they are quite homogeneous in that regard. However, at this stage, we applied the integrated framework to seven cases, and observed a wide variability of capacities within each research funding institution. In the United States, the NIH was created in 1930 and cumulates almost 90 years of experience, whereas the United Kingdom’s National Institute for Health Research was the last to be established in 2006, from the evolution of a previous agency. Canada’s CIHR operated on around US$ 800 million in 2017–2018 (equivalent to over CAN$1 billion), whereas Singapore’s National Medical Research Agency mobilises about half that budget, at US$ 492 million in 2016, leading to a population equivalent of approximately one-seventh that of the Canadian one. Having highly variable internal capacity and yet still portraying a similar set of governance dimensions reinforces the strength of the framework, especially its governance functions. Following a similar line of reasoning, all seven cases operate in diverse national environments and still present consistency through the presence of the five governance functions. Altogether, we argue that the variability of cases reinforces the validity of the governance functions.

Another issue that might arise is that selected institutions might not make it possible to portray the extent of the dimensions of governance at stake. The dimensions first come from the review of frameworks in use, which were then put to the test on seven cases. Notice that we do not intend here to claim that one funding institution is doing a better job than another, or to compare across cases; the highlight is on dimensions, not cases. Any initial dimension that was irrelevant can be expected to be absent from cases, though this was not observed herein. All five governance functions were indeed mentioned by all seven cases. Additionally, one could argue that, initially, we might have missed a dimension important to governance, which is conceptually correct. Furthermore, the analysis of cases would not have made it possible to identify extra dimensions in an easy way as we did not look for a specific additional dimension, nor might such an extra dimension be easily identifiable through documentary analysis. Thus, the test of the governance functions on seven cases could invalidate a dimension if it were to be absent in one or more cases (especially for institutions outside Canada, Australia and the United Kingdom that were also feeding the review of the frames), and it could temporarily validate the importance of an initial dimension that was present in all cases, yet it cannot validate the extent of the governance functions.

Note that this study by no means provides an exhaustive list of HRG settings and mechanisms in selected countries, nor does it compare which funding institutions perform best. Additionally, the intent of this analysis of actionable functions is to identify pragmatic actions under the dimensions (only in terms of governance) of the framework rather than to assess the same institutions on these dimensions.

Although research is ultimately undertaken by researchers in public or private organisations, universities, institutes and centres, we do not intend to provide a framework for institutions hosting research projects, for example, organisations such as the Saskatchewan Health Research Foundation, which recently published a governance framework and policies, mainly for its board.

## Conclusion

Two main contributions come out of this work. First, we bring a conceptual contribution for scholars in the field of governance and health research. We developed an encompassing framework for the governance of health research by national funding institutions. The framework contains 13 functions, wherein 5 are dedicated to governance, 3 dedicated to management, and 5 dedicated to transversal principles that apply to both governance and management. The framework grew out of the combination of existing governance frameworks for health research funding institutions. Second, we bring a practical contribution for high-level managers in charge of governance of health research funding institutions. The framework was broken down into operational dimensions of governance to render the governance function of the framework more actionable. The operational dimensions are extracted from a multiple-case study of seven selected health research funding institutions from North America, Europe and Asia, and the specific actions they put in place to exercise their governance, especially regarding intelligence acquisition, strategy formulation, resourcing and instrumentation, management of relationships, and accountability and performance.

The framework is useful in several ways, namely to point out low-level governance and to track, measure and forestall it. In a sense, pointing out low-level governance can help funding institutions by illuminating whenever one or more functions are given little to no attention. An institution that does not manage partnerships in a diverse and efficient way, seeking out inputs from one or two key players in the private sector, for instance, will be poor at answering the health challenges of its population. It will not perform as well as an institution with open processes that feed the debate as to which challenges must be addressed in the health sector and other sectors that determine the health of the population. Though one institution might, at its inception, choose to focus on one privileged relationship with a specific national partner, governance maturity towards more encompassing actions for improving health through research will, in the long run, rely on a more diverse set of partnerships.

The framework can help in tracking the maturity curve of governance for an institution. Take, for instance, an institution willing to shift gears towards stronger influence in health research – surely tightening ties with partners or focusing funding and exploring wider funding contributors would be an option. The framework could be starting material for performance measurement on the institution’s governance. It could help to develop indicators on each function so that a board can follow-up changes in governance style – putting more or less emphasis on intelligence acquisition or on accountability, or else putting more or less emphasis on some more operational aspects of governance, for instance, acquiring intelligence from institutions’ top influencers, such as politicians, or else making sure citizens get a stronger voice in the governance discussion of institutions. Finally, the framework can be of use to forestall unwanted shifts in governance. Being aware of the current type of governance of the institution, leaning more or less towards one function or another, being more or less prone to the top-down or bottom-up influence of outsiders, for instance, merely implies the institution could take measures against travelling down a road it did not intend to take.

What is left to be done regarding governance of health research funding institutions? We suggest four avenues. Governance does not stand alone as a single action that high-level managers run. Governance is underpinned by principles, or in other words, by what it means for those institutions to operate ‘good’ governance. We suggest those principles are ethics, transparency, capacity reinforcement, monitoring and evaluation, and public engagement. These compose the underlying know-how that applies to either governing or daily management. Further investigation is needed into what it means, in operational terms, to engage the public in accountability or in resourcing, and the like. Additionally, governance runs hand in hand with daily management. Further thought must be given to the complementarity of governance and managerial functions – what does it mean in operational terms? Additionally, and perhaps more intriguingly or more promisingly for better health research, what are the operational governance actions that are in contradiction with some of these operational management actions in place in funding institutions? Finally, in some countries, provincial research funding institutions are key players in funding research and might or not align with national governance standards. Investigating governance functions and actionable functions for provincial funding agencies is an avenue. The same governance and management functions would likely apply to any organisation across health research. The ways in which each function translates into operations in practice is more likely specific by level.

## Data Availability

The data that support the findings of this study are listed in the Appendix.

## Appendix

### Data sources

Sources for the United States of America:

National Institutes of Health (NIH)-Wide Strategic Plan Fiscal Years 2016–2020.

Website of NIH on strategic plan and mission (consulted June 4th, 2019, at https://www.nih.gov/about-nih/what-we-do/mission-goals).

Sources for Canada:

Canadian Institutes of Health Research (CIHR) Act, S.C. 2000, C.6.

CIHR. 2015. Health Research Roadmap II: Capturing Innovation to Produce Better Health and Health Care for Canadians. Strategic Plan 2014/15–2018/19.

CIHR Annual Report 2017–18.

Website of CIHR on strategic plan and mission (Consulted June 4th, 2019, at http://www.cihr-irsc.gc.ca/e/22754.html).

Sources for Australia:

National Health and Medical Research Agency (NHMRC) Strategic Direction 2015–16 to 2018–19.

National Health and Medical Research Council Act 1992. No. 225, 1992 as amended.

NHMRC Annual Report 2016–2017.

Website NHMRC on strategic plan and mission (Consulted June 4th, 2019 at https://www.nhmrc.gov.au/about-us/publications/nhmrc-corporate-plan-2018-2019#toc__32).

Sources for Singapore:

National Medical Research Agency (NMRC) Translating Research into Better Health Annual Report FY2016.

Website of NMRC on strategic plan and mission.

Website of NMRC Who we are (Consulted June 4th, 2019 at https://www.nmrc.gov.sg/who-we-are).

Sources for Sweden:

Tiessen, J. (2008). Health and Medical Research in Sweden. Observatory of Health Research systems. In (pp. 61). Europe: RAND Europe.

Website of the Swedish Research Agency (Vetenskapsrådet, SRC) on strategic plan and mission (Consulted June 4th, 2019 at https://www.vr.se/english/analysis-and-assignments/research-infrastructure/ess-in-sweden/the-swedish-research-councils-ess-mandate.html).

Stafström, S. Date not available. The Swedish Research Council Overview and current issues. Vetenskapsradet.

Sources for the United Kingdom:

National Institute for Health Research (NIHR). Annual Report 2016–2017. Improving the Health and Wealth of the Nation through Research.

Website of NIHR on mission and vision (consulted June 4th, 2019 at https://www.nihr.ac.uk/about-us/our-themes/).

NIHR. 2013. A brief overview of the National Institute for Health Research.

Sources for The Netherlands:

Website of the Netherlands Organisation for Health Research and Development (ZonMW) on mission and vision (Consulted June 4th, 2019 at https://www.zonmw.nl/en/about-zonmw/policy-priorities/).

Website of International Network of Agencies for Health Technology Assessment about ZonMW (Consulted June 4th, 2019 at http://www.inahta.org/members/zonmw/).

ZonMw. Date not available. The Netherlands Organisation for Health Research and Development. Brochure.

## Abbreviations

CIHR: Canadian Institutes of Health Research
COHRED: Council on Health Research for Development
FGHR: Framework on Governance of Health Research
HRG: Health research governance
NHMRC: National Health and Medical Research Agency
NICE: National Institute for Health and Care Excellence
NIH: National Institutes of Health
